# The importance of qualitative social science research for informed public health policy at local and national levels: insights from a local health district in New South Wales, Australia

**DOI:** 10.3389/fpubh.2025.1575188

**Published:** 2025-05-09

**Authors:** Jennifer White, Kirrilly Thompson, Joanne Talwar, David N. Durrheim

**Affiliations:** ^1^Health Protection, Hunter New England Local Health District, New Lambton, NSW, Australia; ^2^School of Medicine and Public Health, University of Newcastle, University Drive Callaghan NSW, Callaghan, NSW, Australia; ^3^Flinders Health and Medical Research Institute, College of Medicine and Public Health, Flinders University, Adelaide, SA, Australia

**Keywords:** public health, qualitative, social science, zoonoses, infection control

## Abstract

Australia employs an evidence-based approach to public health policy, emphasizing disease prevention, health promotion, and healthcare access that is informed by relevant research. However, implementing effective policy can be challenging due to the complexity of various public health issues. Social and behavioral factors significantly impact individual and community health outcomes, necessitating a deeper understanding of their interrelationships. Social science theories and methodologies provide critical insights into the complex relationships between individuals and society. This perspective paper highlights the critical role of qualitative social science research in shaping public policy by offering rich, contextual insights that quantitative data alone cannot capture. This article examines the benefit of incorporating qualitative social science research in a New South Wales (NSW) local health district’s pursuit of evidence-based approaches. Through five case studies, we demonstrate how qualitative social science research has been instrumental in addressing key public health challenges, particularly in managing zoonotic diseases and pandemics, ultimately informing and shaping public health policy.

## Introduction

Best practice public health policy is underpinned by evidence-informed decision-making, which relies on the collection and appraisal of the best available evidence to deliver effective and equitable health services (1–3). Public health policy is inherently complex due to the interplay of multiple factors influenced by government institutions, political ideologies, public opinion, economic conditions, expert research, media, international factors, and cultural context (4). To investigate social phenomena, social scientists employ both quantitative and qualitative methods in their research, or mixed-methods approaches to gain a more comprehensive understanding of social issues.

Traditionally, public health policy has relied heavily on recommendations derived from quantitative data to guide decision-making, evaluate impact, and justify interventions. Governments and organizations use such data to ensure policies are measurable, evidence-based, and outcome-driven. However, in recent decades, qualitative research in social science has played an increasing role in translating evidence into public health policy (5). Qualitative research provides valuable insights into the social, cultural, and behavioral factors that influence health outcomes, interventions, and policies. Indeed, professional experience, expert opinion, patient values, and local contextual factors are all essential contributors to evidence-informed decision-making (6). Moreover, qualitative social science research can guide the development of tailored interventions that resonate with target populations. This includes gaining an understanding of unpredicted relationships, unanticipated outcomes, problematic attitudes, and common barriers and facilitators that may impact the adoption of new approaches. Such findings can assist decision makers in fashioning policy implementation that will have a positive impact at population level (7). Qualitative social science at the local level is essential for creating context-specific public health interventions that address community needs, are socially and culturally acceptable, promote equity, and enhance well-being. By focusing on human behavior and social structures, qualitative social science approaches help ensure that policies and programs are not only evidence-based but are aligned with the lived experiences of the people they aim to serve.

In an Australian context, public health networks and public health units in local health districts (organizations, agencies, and individuals working together to improve the health of a population) are vital players in the public health landscape, serving as a frontline for health service delivery and community engagement. In this article, we consider the ways in which qualitative social science research has influenced public health policy and practice at state and national levels by examining projects led by a public health unit in New South Wales (NSW) that has embraced qualitative social science approaches. As described elsewhere (8), the Hunter New England Local Health District (HNELHD) is located in northern NSW. Situated on the east coast of Australia, it is a diverse area covering 130,000 km. The population of 950,000 includes 65,000 Aboriginal and Torres Strait Islander people (9). Along the coastal margins, population density is reasonably high with concentrated urban development. Large national parks and untouched woodland cover the Great Dividing Range, a rugged area parallel and approximately 200 kms inland. In the easterly formations of the Great Dividing Range lies the Hunter Valley, home to Australia’s oldest wine growing region and the second-largest thoroughbred horse breeding region in the world, combined with a long history of industrial and mining activity. To the immediate west of this Range is fertile agricultural land with many small towns. The remote western areas comprise arid country suited to low-intensity livestock-farming and discrete First Nation settlements (First Nations refers to Aboriginal and Torres Strait Islander people).

Major public health issues for the HNELHD over recent years have included infectious disease threats, including zoonoses, and the need to address disparities in the health of First Nations people (10). In this article, we present case studies that illustrate the flexibility and diversity of qualitative social science approaches successfully applied by the Hunter New England Local Health District Public Health Unit (HNELHD PHU) in partnership with the University of Newcastle, to inform local, state and national policy. The partnership between the HNELHD PHU and the University of Newcastle has developed over more than a decade, grounded in a shared recognition of the importance of addressing complex public health challenges through research, including qualitative social science. The need for such a partnership was highlighted by the recognition that public health interventions and responses are often more effective when they draw upon the expertise of social sciences, health promotion, epidemiology, environmental health, and other related fields. The partnership is structured around mutual respect, regular communication, and a commitment to co-design, with researchers and public health practitioners working together from project inception to result implementation and evaluation. This includes mutually accepted ethics submissions, shared project governance, embedded research roles within the health service, and capacity-building initiatives that strengthen qualitative research skills among health staff. The qualitative social science approaches for consistency used in the presented case studies include semi-structured interviews, focus groups (group meetings) and participatory action research (PAR).

## Case study 1: personal biosecurity in the equine industry (11, 12)

People working in the NSW equine industry are risk of exposure to zoonoses, ranging from ringworm to the potentially fatal Hendra virus (HeV) infection. The risk of equine zoonoses is heightened for those working in the breeding industry, as foaling and foal-handling activities expose workers to risks such as equine chlamydiosis (13). In 2018, the HNELHD PHU and the University of Newcastle conducted a study to explore the uptake of personal biosecurity and infection prevention and control (IPC) measures among professionals in the equine industry. The research involved interviews and focus groups with 29 participants, including veterinarians, veterinary nurses, foaling staff, stud managers, and laboratory personnel working in NSW, Australia (11). This led to the identification of various social and physical factors impacting infection control and personal biosecurity practices. Findings were fed back to 17 participants (representing 14 thoroughbred breeding farms and three equine veterinary practices), who subsequently identified 16 unique personal biosecurity strategies that were trialled in ensuing research (12). The strategies encompassed personal protective equipment (PPE), zoonotic disease awareness, policies and protocols, supportive environments, and leadership. Supportive materials were provided, including posters about zoonoses, using a “PPE buddy,” an equine stud action plan outlining the 16 strategies, a poster presentation on using the “if - when - then” heuristic in action (14), zoonotic risk education and exposure pathways, a tiered approach to PPE, and hand hygiene. Strategies were trialled by participants, and the uptake was monitored through three repeat surveys and supported by regular optional online meetings facilitated by an industry champion. Additionally, follow-up interviews were conducted, where participants reviewed survey results and elaborated on strategy uptake. Finally, a workshop was held in Scone—the heart of Australia’s thoroughbred breeding industry—to present the final findings and disseminate practical educational materials. This project demonstrates the value of in-depth exploration of issues relevant to the local community and the benefit of close engagement and communication between workers, industry, HNELHD PHU and the University of Newcastle. Involving all partners in research design, leadership, interpretation and application, fostered effective and trusting relationships that proved invaluable for designing ongoing public health advice, response and implementation.

## Case study 2: Navigating experiences and risk perceptions during Hendra virus investigations (15)

In 2022, a novel HeV genotype was detected in a horse in Newcastle, NSW (15). A published report was essential to communicate this development, given the potentially fatal human consequences without appropriate management. HeV is considered a One Health issue because it affects the health of animals and humans, with key environmental determinants, requiring a collaborative, multisectoral, and multidisciplinary approach to manage and prevent its spread (16). As part of the local One Health response involving a multidisciplinary team, a case description was prepared involving 15 experts from across human, animal and environmental health. The social aspects of the human-horse relationship in this case study were also noted, including the profound stress of public health unit responses and grief following the sudden loss of a loved horse. In 2024, an in-depth qualitative research project was designed within a social science framework of interpretive phenomenology to further understand how horse owners and veterinarians personally experienced a horse with HeV and how public health can better respond. Interviews with horse owners (*n* = 8) and veterinarians (*n* = 5) in a regional setting of northern New South Wales, Australia, captured the challenge of negotiating the complex scenario of identifying and responding to HeV; pervasive affects and grief responses; and implications for future public health responses. Gaining an understanding of the personal experience of owners and veterinarians of a rare but potentially high consequence event provided insights into how public health services can better respond to support owners to adopt protective behaviors (17). In particular, the research identified the need for enhanced grief support, detailed advice for managing a deteriorating situation, and for public health liaison with trusted general practitioners to facilitate education and management. As a result of this work, the Australian guidelines for public health responses to HeV have been updated. Overall, this research highlighted the importance of social science in the One Health armamentarium for understanding lived experiences and informing the management of emerging zoonotic diseases, such as HeV.

## Case study 3: responding to human-bat interactions (18)

Australian bat lyssavirus (ABLV) infects flying fox and insectivorous bat species in Australia. Zoonotic disease transmission to humans has a fatal outcome and prior vaccination and/or post-exposure treatment (PET) is required. In Australia, surveillance data from 2007 to 2011 revealed a four-fold increase in the number of people receiving PET despite public health messaging about the risks associated with bat contact. An interview based study was conducted with 16 individuals with non-occupation related potential ABLV exposure (bat scratch or bite), identified from the NSW Notifiable Conditions Incident Management System, in the HNELHD (between July 2011 and July 2013) (18). Findings explained how, despite awareness of disease risk, a deep concern for bat welfare was a key driver in rescuing animals that were caught in fencing or appeared to be unwell. As a result of this research, an important change was made to NSW public health messaging from a focus on “any contact with a bat should be avoided” to “if you handle or touch a bat, you could do it harm.” Instead, people were encouraged to contact registered animal welfare services (with details provided), since they are trained to handle bats correctly. In response, NSW Wildlife Information, Rescue and Education Service (WIRES), an Australian Wildlife Rescue Organisation in NSW, anecdotally reported an increased number of calls from community members who had found trapped or languishing bats requesting their assistance rather than handling the animals themselves.

## Case study 4: community voices and community control for pandemic containment in First Nations communities in Australia (19, 20)

The experience of past pandemics has highlighted the importance of inclusive public health strategies, particularly for First Nations populations (21). Social disparity, institutionalized racism within health services and differences in access to culturally safe health services contribute to disadvantage and delayed appropriate treatment (22). The importance of a First Nations focus had not been reflected in the Australian pandemic plan prior to the 2009 H1N1 influenza pandemic. In Australia, in 2009, the H1N1 influenza pandemic effected First Nations populations more than non-First Nations populations (23). A research team comprised of academics, First Nations and non-First Nations people from a wide variety of disciplines including medicine, veterinary science, epidemiology, public health, anthropology, health promotion, nursing, and education was developed, and ethics across jurisdictions and universities was obtained. This national study facilitated by the HNELHD PHU team subsequently explored the perceptions of Aboriginal and Torres Strait Islander people (community groups, organizations) and their experiences with H1N1 using a qualitative PAR framework (20). PAR involves direct collaboration with those affected by an issue to understand and improve upon practices for the purpose of action or change (24). Forty-seven interviews and 10 focus groups (with an average of five participants per focus group) were completed from July 2009 to May 2010. Key themes identified were the importance of family; ways of life, and realities of living in response to influenza; and key messages to government and health services to focus on communication, understanding and respect. A report provided guidance to health services and other relevant organizations to improve their responses to pandemic influenza; however the National Action Plan for Human Influenza Pandemic (NAP) failed to mention Aboriginal and Torres Strait Islander people (25). Australia’s Closing the Gap strategies (26) have increased in importance since this time, and learnings from this study were successfully used to advocate for the explicit prioritization of Aboriginal and Torres Strait Islander peoples in the National Pandemic Plan during the COVID-19 pandemic response. Specifically, the HNELHD PHU actioned these findings by embedding cultural governance and leadership in the COVID-19 pandemic response (27). This ensured effective containment and elimination of the Alpha and Delta SARS-CoV-2 waves within HNELHD Aboriginal communities through intensive, culturally informed practical support for isolation of cases and quarantine of contacts, and informed communication strategies, and immunization efforts.

## Case study 5: tailoring immunization programmes in Maitland, New South Wales (28, 29)

In 2017, it was identified that the regional center of Maitland, NSW had high numbers and rates of children who were overdue for scheduled vaccinations (2016, *n* = 344, 37.7%). In response, the HNELHD PHU implemented the World Health Organization Tailoring Immunisation Programme (TIP) (30) to promote vaccination. TIP uses quantitative data collection methods to identify areas of low coverage within a population, and qualitative methods to explore barriers and drivers for vaccination in that community. The program is underpinned by the Capability, Opportunity, and Motivation (COM-B) model of behavior change (28). COM-B considers individual factors influencing behavior—such as capability and motivation—as well as contextual factors, including opportunity, which may be social (e.g., cultural norms, support) or physical (e.g., access to services) (28). The final phase of TIP involves co-designing a tailored approach to achieve high and equitable vaccination uptake, regardless of factors such as income, education, geography, and cultural background. This is followed by ongoing monitoring and evaluation of the strategy.

As part of implementation of the TIP initiative in Maitland (referred to as Maitland TIPs), the project began with an in-depth analysis of coverage data from the Australian Immunization Register—a national system that records vaccinations administered to individuals across Australia. This quantitative review was a careful exploration, at the most granular administrative level available over time, to identify specific demographic sub-groups that were missing out. It was followed by a qualitative phase involving 34 in-depth interviews and six focus groups to explore community immunization perspectives and experiences in greater depth. In total there were 25 participants, including parents and service providers, gaining a deeper understanding of the factors influencing immunization (29). Key themes were (i) limited engagement with health services unless the need is urgent, (ii) multi-dimensional access barriers to immunization services in Maitland, (iii) a flexible, supportive family centered, primary health care approach, utilizing strong partnerships, is most likely to be effective in increasing childhood immunization rates in Maitland, (iv) data can be used more effectively to inform service providers about trends and individual children not fully immunized (29). A tailored strategy was subsequently co-designed by parents and health service providers, incorporating friendly, personalized reminders, outreach appointments, and home visits for families most in need (29). A process evaluation of Maitland TIPs found that coverage rates increased from 62.3% (2016) to 86.2% (2020) (30). This success demonstrates the effectiveness of the TIP approach in improving childhood immunization coverage. As a result, TIPs is now being implemented in other communities in NSW with low vaccination coverage.

## Discussion

This article presents five case studies that demonstrate the benefits of applying qualitative social science approaches to gain an in-depth understanding of public health issues, thereby supporting the development of effective interventions, particularly within local communities. The qualitative social science approaches used in these case studies had significant impacts on public health responses by providing in-depth insights into the personal experiences, behaviors, and motivations of the people directly affected by zoonotic diseases and other public health challenges. These methods helped researchers and practitioners identify barriers, needs, and opportunities that would not have been uncovered through purely quantitative measures. By emphasizing subjective experience, emotional responses, and community dynamics, the research helped shape more effective and culturally appropriate public health strategies. The lessons learned and presented in the case studies have global relevance, especially in settings with diverse populations and complex public health challenges. We posit that the integration of qualitative social science approaches, community engagement, and co-designed interventions can be adapted to various international contexts to improve public health outcomes.

This paper highlights the strengths of qualitative social science in public health by providing context-rich, real-world insights that help shape policies, interventions, and service delivery. Central to this study was the implementation of study designs that were underpinned by theoretical frameworks, involved multiple data sources and triangulation of qualitative and quantitative data. While providing rich contextual details promotes transferability of findings, a limitation of each case study is the modest sample size and potential for reduced generalizability of findings. We also acknowledge the lack of long-term follow-up of data to assess the sustainability and long-term impact of the implemented strategies. Further applied research in the local context is needed, involving larger-scale and longer-term studies to validate and refine these approaches. Nonetheless, a key strength of the presented case studies was having a community-centered approach, involvement with partners, a focus on behavior and experience, and holistic multidisciplinary approaches that all resulted in an improvement in public health messaging, practice and policy. Indeed, the principle of involving communities in the design and implementation of public health strategies is highly applicable worldwide. Even in regions with different social structures, health systems, or cultural beliefs, understanding local cultural, social, and behavioral contexts is crucial to successful health interventions.

## Conclusion

The use of qualitative social science approaches in these case studies revealed deeper insights into human behavior and emotions, which are often overlooked in traditional quantitative research. The common threads running through the studies—community engagement, relationship building, understanding of behavior, and the improvement of health communication—demonstrate the critical role that qualitative research plays in developing more effective, tailored, and culturally appropriate public health responses. The integration of these findings into public health policy and practice enhances the relevance and impact of interventions, ensuring they address real-world challenges and resonate with the communities they aim to protect.

## Data Availability

The data supporting the conclusions in this study comprise multiple embedded case studies and are available by contacting the relevant corresponding author on reasonable request.
